# Continued Root Formation after a Compound root Fracture in an Immature Permanent Incisor: A Case Report with a 2-year follow-up 

**DOI:** 10.30476/dentjods.2024.104235.2512

**Published:** 2025-09-01

**Authors:** Maryam Enteghad, Safoora Sahebi, Saleh Hadian

**Affiliations:** 1 Postgraduate Student of Endodontics, Dept. of Endodontics, School of Dentistry, Shiraz University of Medical Sciences, Shiraz, Iran.; 2 Dept. of Endodontics, School of Dentistry, Shiraz University of Medical Sciences, Shiraz, Iran.

**Keywords:** Traumatology, Tooth Fracture, Tooth Discoloration, Endodontics

## Abstract

While traumatic injuries in the young permanent dentition are frequent, root fractures are relatively rare, particularly in immature teeth. This study reports the case of a 7-year-old boy who fell off a bicycle. Radiographic examination showed an immature right upper central incisor with fractures in the middle and along the root in an oblique and horizontal direction. Furthermore, there was an extrusion of the coronal segment from its original position. At the first appointment, the right central incisor was repositioned, and a semi-rigid splint was applied for four weeks. The patient was examined periodically for the following two years. After two months, the injured tooth was asymptomatic, with a reduction in probing depth from 8 mm to 2 mm along the tooth surface and a physiologic mobility. Although the injured tooth responded to the electric pulp test after nine months, it had no response to the cold test even after two years. The injured tooth showed continued root maturation of both coronal and apical fragments, although metamorphosis calcification and root canal narrowing were observed in conjunction with mild yellow crown discoloration. This report highlights the ability of Hertwig’s epithelial root sheath and immature pulp to continue root development in fractured immature teeth.

## Introduction

The majority incidence of traumatic dental injuries occur in children between the ages of 7 and 12, caused by falls and accidents close to home or school [ 1 ]. The condition mostly affects the anterior teeth, with a greater impact on maxilla compared to mandible [ 2 ]. Horizontal root fracture (HRF), or transverse root fracture, is an uncommon condition, accounting for 0.5% to 7.0% of all injuries in permanent dentition [ 3 ]. It is described as those involving dentin, cementum, periodontal ligaments, and the pulp. The fracture line may be horizontal, oblique, or a combination of both, but typically has an oblique orientation along the root surface. Clinical findings include tenderness, mobility, coronal segment displacement, and bleeding from the gingival sulcus [ 4 ]. The International Association of Dental Traumatology (IADT) guideline states that the tooth's coronal section, if displaced, should be repositioned as quickly as possible and stabilized for four weeks using a flexible and passive splint. In cervically located fractures, prolonged stabilization for a duration of four month may be required [ 4 ]. Pulp necrosis in the apical segment is extremely rare due to the fact that the apical pulpal circulation is not interrupted. However, 25% of cases undergo pulpal necrosis of the coronal section, necessitating endodontic treatment [ 5 ]. 

HRFs frequently happen in teeth with complete root development at the anterior maxilla. Despite the thin and delicate dentinal walls of immature teeth with open apices being susceptible to fracture, it is claimed that such teeth with vital pulps rarely experience HRFs [ 6 ]. While managing immature teeth, the practitioner faces many endodontic and restorative challenges. On the other hand, treatment failure could ultimately result in tooth loss with serious lifetime consequences. So, it is crucial for the practitioners to understand the biological basis, diagnostic approaches and suitable treatment options for root-fractured immature teeth [ 7 ]. 

This case report describes the clinical management of a root fracture in an immature maxillary central incisor with following up its root development for 2 years. 

## Case Presentation

A 7-year-old boy was referred to Shiraz University’s Faculty of Dentistry Department of Traumatology with a complaint of pain, tenderness and mobility in his upper right central incisor as a result of falling down from the bicycle, 3 days before. There were no extraoral symptoms. Intraoral examination revealed neither soft tissue laceration nor any teeth crown fracture. Both central incisors were semi-erupted while the lateral incisors had not erupted yet. The maxillary right incisor showed an extrusion of about 2 millimeters with Glickman’s grade III mobility. The previous splint was performed immediately after the trauma by a general dentist in the patient’s hometown, but it was debonded. Also, both upper centrals were tender to touch and percussion test. A probing depth of around 8 mm in the mesial and palatal surface of right upper incisor was noted. Both incisors had a negative response to pulp sensibility tests (electrical pulp test, cold test) but no color alteration was observed. In the radiographic examinations, periapical radiographs have been provided for all anterior teeth from three different angles. Both incisors had open apices, and presented only one-third of the complete radicular development. The dentinal walls were so delicate and thin. Also, a fracture line was detected in the middle third and along the root of the right upper central incisor in an oblique and horizontal direction. 

Following the diagnosis of HRF, two treatment options were available: (1) repositioning and splinting of the fractured tooth and hoping for the best, and (2) extracting the tooth, which could be a more definite treatment plan based on its questionable prognosis, but it undoubtedly could cause a vertical alveolar bone defect and other complications. So, we chose the first treatment option. 

The treatment was initiated by removing the previous insufficient splint, repositioning the coronal fragment with finger pressure and applying a fixed semi-rigid splint (0.4mm round Ni-Ti wire and flowable composite) under local anesthesia. The semi-rigid splint was extended to the primary canines bilaterally to provide adequate stabilization. The correct position of the tooth was confirmed by a periapical radiograph. The splint was retained for 4 weeks. Also, occlusal adjustment was performed in the first appointment, and during the follow-up periods. Gentle brushing, soft diet, rinsing with chlorhexidine gluconate 0.12% mouthwash, and avoidance of chewing on the injured tooth were recommended to the patient. Follow-up examinations were conducted at 1, 2, 4, 6, 12 and 24 months after the trauma. Additionally, informed written consent was acquired from the patient for the publication of this case report and any associated pictures. After four weeks, the resin wire splint was removed. The right central tooth mobility was decreased from Glickman's Grade III to Grade I. After 2months, the probing depth was decreased to 2mm along the tooth surface. The injured teeth were completely asymptomatic and exhibited physiologic mobility

**Figure 1:**
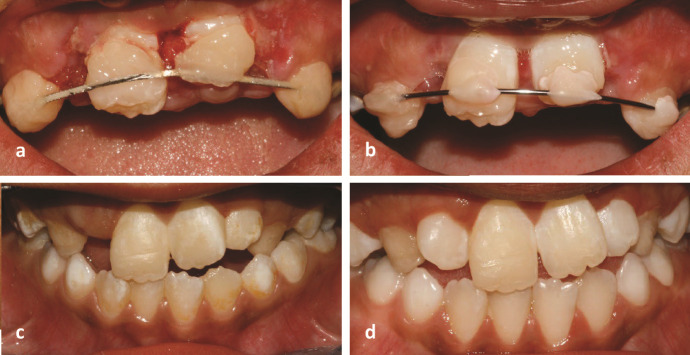
**a:** Extrusion of the coronal fragment and the presence of the previous deboned splint, performed by a general dentist in the patient’s hometown, **b:** Immediately after the repositioning of the displaced tooth, providing a semi-rigid splint extended bilaterally to the primary canines, **c:** One-year follow-up, observation of a mild yellow crown discoloration in the right central incisor, suggesting metamorphosis calcification, **d:** Two-year follow-up

**Figure 2:**
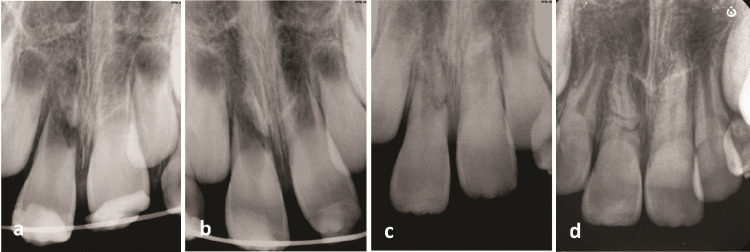
Radiographic examinations, **a:** Pretreatment radiograph indicating an immature right upper central incisor with fractures in the middle and along the root in an oblique and horizontal direction in combination with an extrusion of the coronal segment. Both incisors had open apices, **b:** Immediately after the repositioning, indicating adequate repositioning of the coronal segment, **c:** One year follow-up, the presence of connective tissue healing between the root fragments, both incisors fully erupted, and the development of two-third of the roots was evident, **d:** Two years follow-up, both incisors' canals narrowing and full root development are seen

**Figure 3:**
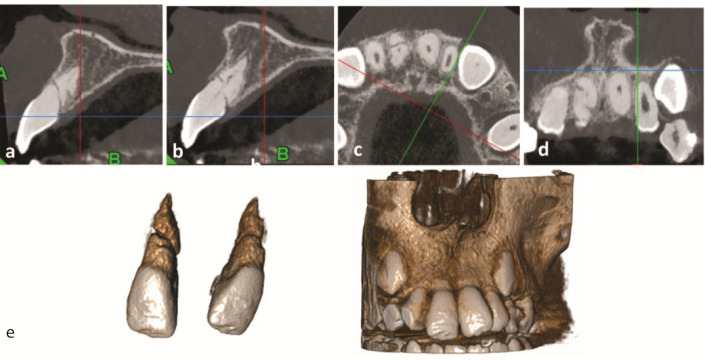
Ultra-low-dose CBCT section; **a and b:** Sagittal view, **c:** Axial view, **d:** Coronal view, **e:** 3D model in the two-year follow-up, showing the presence of an oblique radiolucent line in the coronal third of the fully developed fractured root. The narrow radiolucent line between the fragments and rounding of the fractured edges, indicates the healing type II

Although the fractured tooth responded to the electric pulp test after nine months, it had no response to the cold test even after two years. The left upper central incisor with the diagnosis of concussion, showed a delayed positive response to cold and electric pulp test, 3 months after the trauma. In the 6-month follow-up, radiographs revealed the presence of connective tissue healing between the root fragments. After one year, both incisors fully erupted, and the development of two-third of the roots was evident. Radiographs taken in the 24-month follow-up showed both incisors' canals narrowing and full root development. Additionally, a mild yellow crown discoloration on the left central incisor was observed, indicating metamorphosis calcification (Figures 1-2). Despite the potential benefits of obtaining cone beam computed tomography (CBCT) to determine the fracture’s precise situation, it was not feasible at the initial visit due to the patient's young age and lack of cooperation. Therefore, an ultra-low-dose CBCT was suggested for further examination in the two-year follow-up. Figure 3 shows the presence of an oblique radiolucent line in the coronal third of the fully developed fractured root, with rounded edges, which indicated the healing type II.

## Discussion

Root fractures occur when a tooth experiences a horizontal impact from a hard object or during a fight. Small and sharp objects focus their power on a smaller surface on the tooth, causing fractures rather than dislocations [ 8 ]. They can occur in conjunction with other injuries. Concussions and subluxations are the most common coexisting injuries with root fractures. A precise diagnosis is the initial step in treatment as they might not be noticeable immediately after the injury, particularly if the coronal segment is not displaced [ 9 ]. Therefore, at least three angled periapical radiographs are required for reliable fracture detection. Despite the delicate and thin dentinal walls of immature teeth, they rarely experience root fractures. This report describes an extremely rare situation of a compound fracture in a tooth that the development of just one-third of the root was occurred.

The prognosis and treatment of root fractures depend on the fracture location. Apical fractures often require minimal intervention and have a favorable prognosis, whereas coronal and supracrestal root fractures are more susceptible for bacterial contamination, requiring complicated management. Additionally, fractures in the cervical region are more commonly horizontal, whereas those in the apical or mid-root regions tend to be more oblique [ 8 ]. The fracture lines in our case had oblique orientations, although it was positioned at the middle of the root. It should be noted that although the fracture line was first located in the middle third of the short root in the first visit, subsequent root development during a two-year follow-up period led to the fracture location shifting to the coronal third. 

When a root fracture happens, the displacement of the coronal segment could stretch or lacerate the dental pulp, leading to a decrease in blood flow and pulp necrosis in the coronal segment. If there is a way for bacteria to penetrate the tooth, an infection might develop in the root canal. In the present case, the fracture line was located at the middle third of the root, and there was a noticeable deep pocket on the mesial and palatal surfaces, which may indicate a potential risk of pulp contamination [ 5 ]. Nevertheless, the probing depth was reduced during the follow-up period, and the pulp did not experience necrosis. The apical fragment is not typically affected by the injury as the impact forces are fully absorbed at the fracture site, so the pulp in this segment remains vital [ 5 , 10 ]. In our patient, root maturation and apex closure persisted even after the root fracture. Several reasons could explain this phenomenon. First, Hert-wig's sheath in the unaffected apical segment has survived and maintained its capacity to organize root development. Second, the immature pulp tissue with sufficient blood circulation and potent stem cells could efficiently eliminate the inflammation and continue dentin formation. 

A root fracture can also trigger a complex chain of injuries to the surrounding hard tissues, with the thin labial wall of the alveolar socket being the most susceptible to fracture [ 9 ]. Nevertheless, in this patient, the buccal plate bone was intact, and the malalignment of the central incisors were resulted from their labial eruption rather than being caused by the injury. Luckily, there was enough attached gingiva surrounding the tooth. According to the updated guidelines, the fractured root should be monitored for two years prior to orthodontic movement [ 11 ]. After the observation of proper healing in two years by employing CBCT scans, the patient was referred to an orthodontist for subsequent esthetic treatment. 

At the first appointment, clinical examination should include all routine pulpal and periapical tests. Temporary loss of sensibility is a common finding, particularly after luxation injuries. Thus, the absence of response to the pulp sensibility test is not clear evidence of pulp necrosis, as was seen in our case [ 12 ]. Antibiotics are not recommended for teeth during the stabilization period, except if the coronal segment has been avulsed. In our case, the antimicrobial therapy was limited to the use of chlorhexidine gluconate mouthwash two times a day.

The healing of HRFs is influenced by various factors such as age, location of fracture, dislocation severity, splint type and duration, and treatment delay. Andreasen and Hjorting–Hansen classified fracture line radiographic healing into four types (I-IV) [ 9 ]. In the presented case, healing type II with connective tissue took place in the fractured area, radiographically observed as narrow radiolucent line between the fragments and roun-ding the fractured edges in CBCT sections (Figure 3). 

Although the patient was referred to a dentist in his hometown soon after the accident, the initial fixation performed was not appropriate and it was replaced three days after. This might be the reason of pocket formation and connective tissue healing. Fortunately, the pulp initially recovered from the accident and continued to function with root maturation and pulp canal calcification. However, long-term follow-up is required to avoid undesired outcomes.

Pulp canal calcification is fairly prevalent after root fractures ranged from 69% to 73%. This calcification is considered a positive sign of pulp survival. It is a normal physiological response, and there is no indication for endodontic treatment. Nevertheless, calcification reduces the conductivity of temperature changes throughout the tooth. So, cold testing is not as reliable as electric pulp test, as was seen in this case [ 10 ]. Additionally, approximately 60% of teeth with root fractures will exhibit various forms of root resorption. However, it usually has no clinical significance because most of them are just transient surface resorptive processes, resulting in rounded corners at the fracture site in both the coronal and apical fragments [ 10 - 12 ]. Fortunately, minimal resorption occurred in this injured tooth. Finally, it should be noted that informed written consent was acquired from the patient for the publication of this case report and any associated pictures. 

## Conclusion

It is concluded that even the compound root fractures in immature teeth can have a chance for proper healing and root development can proceed if the pulp vitality is maintained. The application of splints alone is effective under appropriate conditions for treating root fractures in immature teeth. Nevertheless, long-term monitoring of trauma patients is crucial as pathologies may manifest several years post-injury
